# Are HFOs in the Intra-operative ECoG Related to Hippocampal Sclerosis, Volume and IQ?

**DOI:** 10.3389/fneur.2021.645925

**Published:** 2021-03-24

**Authors:** Paula Agudelo Valencia, Nicole E. C. van Klink, Maryse A. van ‘t Klooster, Willemiek J. E. M. Zweiphenning, Banu Swampillai, Pieter van Eijsden, Tineke Gebbink, Martine J. E. van Zandvoort, Maeike Zijlmans, The RESPect Database Study Group

**Affiliations:** ^1^Department of Neurology and Neurosurgery, University Medical Center Utrecht Brain Center, University Utrecht, Utrecht, Netherlands; ^2^Stiching Epilepsie Instellingen Nederland (SEIN), Heemstede, Netherlands

**Keywords:** high frequency oscillations, epilepsy surgery, mesial temporal lobe epilepsy, hippocampal volumetry, cognition

## Abstract

Temporal lobe epilepsy (TLE) is the most common form of refractory focal epilepsy and is often associated with hippocampal sclerosis (HS) and cognitive disturbances. Over the last decade, high frequency oscillations (HFOs) in the intraoperative electrocorticography (ioECoG) have been proposed to be biomarkers for the delineation of epileptic tissue but hippocampal ripples have also been associated with memory consolidation. Healthy hippocampi can show prolonged ripple activity in stereo- EEG. We aimed to identify how the HFO rates [ripples (80–250 Hz, fast ripples (250–500 Hz); prolonged ripples (80–250 Hz, 200–500 ms)] in the pre-resection ioECoG over subtemporal area (hippocampus) and lateral temporal neocortex relate to presence of hippocampal sclerosis, the hippocampal volume quantified on MRI and the severity of cognitive impairment in TLE patients. Volumetric measurement of hippocampal subregions was performed in 47 patients with TLE, who underwent ioECoG. Ripples, prolonged ripples, and fast ripples were visually marked and rates of HFOs were calculated. The intellectual quotient (IQ) before resection was determined. There was a trend toward higher rates of ripples and fast ripples in subtemporal electrodes vs. the lateral neocortex (ripples: 2.1 vs. 1.3/min; fast ripples: 0.9 vs. 0.2/min). Patients with HS showed higher rates of subtemporal fast ripples than other patients (*Z* = −2.51, *p* = 0.012). Prolonged ripples were only found in the lateral temporal neocortex. The normalized ratio (smallest/largest) of hippocampal volume was correlated to pre-resection IQ (*r* = 0.45, *p* = 0.015). There was no correlation between HFO rates and hippocampal volumes or HFO rates and IQ. To conclude, intra-operative fast ripples were a marker for HS, but ripples and fast ripples were not linearly correlated with either the amount of hippocampal atrophy, nor for pre-surgical IQ.

## Introduction

Temporal lobe epilepsy (TLE) is the most common epileptic syndrome of focal refractory epilepsy and is subcategorized in neocortical and mesial temporal epilepsy (MTLE). MTLE is often associated with hippocampal sclerosis (HS) (1). Neurosurgery is a therapeutic option for patients with focal refractory MTLE, with a high chance of seizure freedom (2, 3).

Intraoperative electrocorticography (ioECoG) can be used to demarcate the epileptogenic tissue and guide the neurosurgeon in verification whether the hippocampus is affected and the extent of the temporal resection that is required (4).

High frequency oscillations (HFOs) (ripples: 80–250 Hz, fast ripples: 250–500 Hz) are a new electrophysiological biomarker in the ioECoG (5). Ripples and fast ripples have been identified at the seizure onset zone, occurring both interictally and ictally, suggesting a relationship with the mechanisms of seizure onset (6). However, relatively long periods of high amplitude ripple activity occurring in healthy hippocampi were not associated with epilepsy and may relate to physiological brain functioning (7, 8). For this reason, recently a new type of HFO has been proposed: prolonged ripples, described as a ripple event lasting more than 200 ms (9, 10). Nonetheless, the differentiation between physiological and pathological HFOs in the hippocampus remains challenging and the overall significance of this biomarker is unclear.

MTLE, and HS in particular, is strongly associated with memory and cognitive impairment (11). The degree of hippocampal atrophy in HS is negatively associated with memory loss and IQ (12, 13). We compare fast ripples, ripples and prolonged ripples in subtemporal strip electrodes and lateral neocortical electrodes to hippocampal volume, HS and IQ. We hypothesize that increased fast ripples and decreased prolonged ripples are associated with a reduced hippocampal volume on MRI and are related to the severity of IQ impairment in MTLE.More specifically, we expect an increased rate of pathological HFOs, i.e., fast ripples and short ripples and a decreased rate of prolonged ripples to be associated with reduced hippocampal volume and IQ score. This study will provide insight on the relationship between electrophysiology, pathology and cognitive function in TLE, in an attempt to enable prediction of the effects of removing the hippocampus on seizure outcome and cognitive functioning.

## Materials and Methods

### Study Population

**Figure 1:**
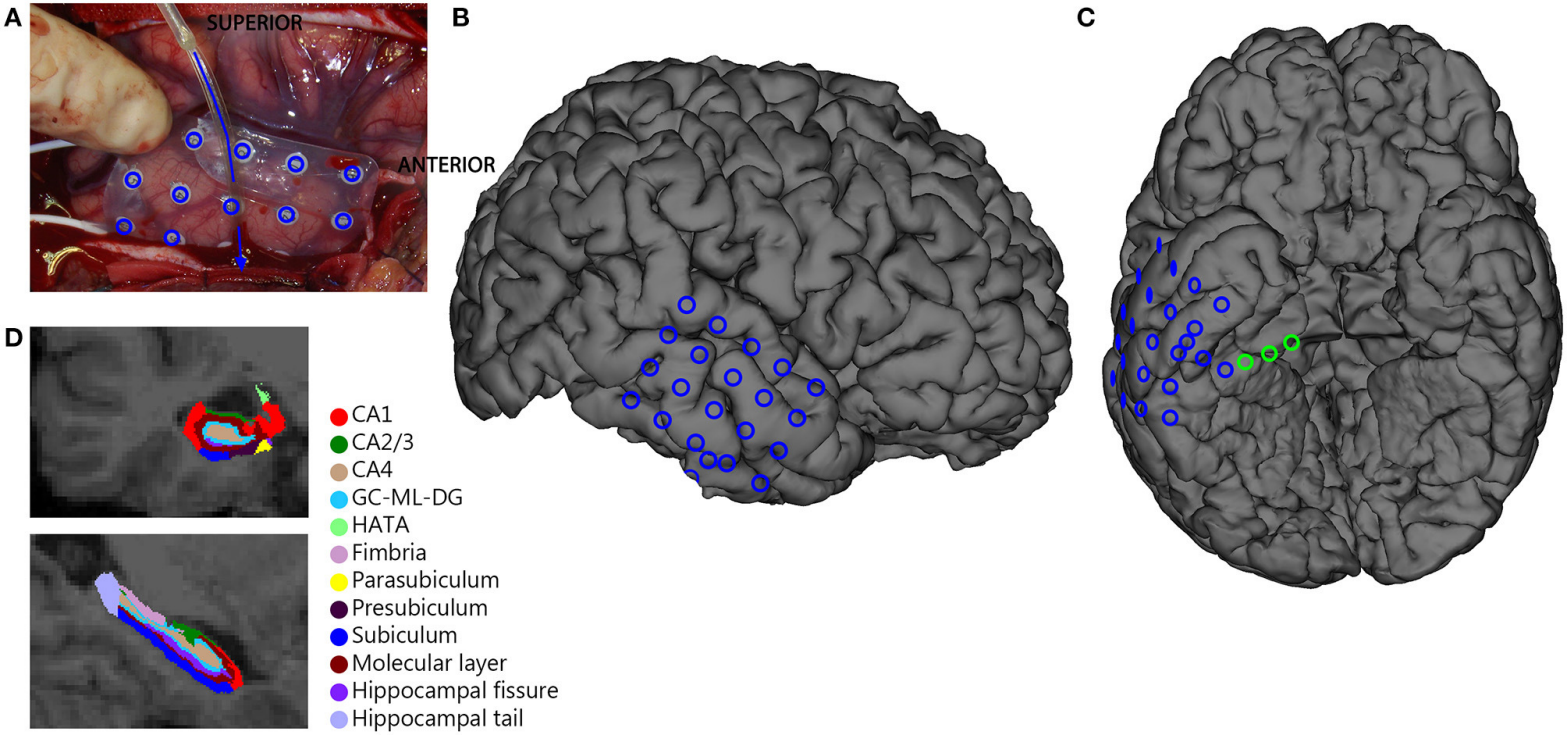
Overview of ioECoG grid placement in anterior temporal lobe surgery. A 4x5 grid is placed on the lateral convexity of the right anterior temporal lobe (blue in **A–C**), and a 1x8 strip is placed under the base of the temporal pole (arrow in **A**)The three deepest contacts of the subtemporal strip sample the hippocampus and the entorhinal cortex and are analyzed separately (green in **C**). **(D)** Example of Freesurfer segmentation of hippocampal subregions.

People who underwent surgical resection of the hippocampus in the UMC Utrecht between 2008 and 2017 were selected from the RESPect database (**R**egistry for **E**pilepsy **S**urgery **P**atients in the UMC Utrecht). Patients were included when they had a diagnosis of MTLE, underwent ioECoG with a sampling frequency of 2,048 Hz, and had surgical resection of the hippocampus. Only patients with subtemporal strip electrodes recording the entorhinal cortex of the parahippocampal gyrus (aimed at recording the hippocampal activity) available for HFO analysis (Figure 1) and a pre-operative 3DT1 and FLAIR or T2 MRI available for hippocampus volumetry were selected. We excluded patients with dual pathology and continuous burst suppression on the ioECoG. We determined if the side of surgery was in the dominant hemisphere, using clinical information on handedness and results from fTCD, fMRI and Wada tests. In our center, no language lateralization test is performed if the planned resection does not include possible Wernicke's areas.

The Medal Ethical Committee of the UMC Utrecht waived the need for informed consent for all retrospectively collected data before 2018 and approved the use of coded data in the RESPect database for retrospective research.

### Pathology

Pathology findings were classified into five categories: HS, central nervous system (CNS) tumors (incl. DNETs), malformations of cortical development [incl. focal cortical dysplasia (FCD)], other (incl. cavernoma, vascular abnormalities, gliosis) and no abnormalities. Subsequently, patients were dichotomized in two groups based on pathology: HS group (ILAE type 1, 2, or 3) and non-HS group (pathological results from hippocampus showing normal tissue or neurons with reactive gliosis only).

### MRI Acquisition

The clinical pre-surgical MRI scans were performed in the UMC Utrecht with Philips MRI-scanners with a protocol designed for epilepsy patients. The parameters of the sequences, the field strengths and the planes changed over time and could be different amongst patients. This study includes 1T, 1.5T, 3T, and 7T scans. All patients had a 3D T1 scan, with a maximum isotropic resolution of 1 mm. T2 scans could be 3D T2, 3D FLAIR, or FLAIR scans and T2 scans in axial or sagittal plane. The images were saved as DICOM files and converted to Nifti for further analysis.

### Hippocampal Volumetry

Image processing and volumetric measurement of hippocampal subregions was done using FreeSurfer image analysis (version 6.0). An automated segmentation of the hippocampal subregions was performed based on a 3D T1-weighted scan and a 3D FLAIR sequence. In case there was no 3D FLAIR available, a FLAIR sequence was used and in absence of a FLAIR any available presurgical T2-weighted scan with the highest resolution was used. Volumes of the following subregions were calculated: CA1, CA3, CA4, subiculum, presubiculum, parasubiculum, granule cells in the molecular layer of the dentate gyrus (GC-ML-DG), hippocampal tail, fimbria, hippocampal amygdaloid transition area (HATA), hippocampal fissure and the total hippocampus. The hippocampal segmentations in different planes were reviewed for correctness in the Freesurfer imaging software (Figure 1D, Freeview; https://surfer.nmr.mgh.harvard.edu/). All volumes were corrected by division over the total intracranial volume to be able to compare across patients, therefore all volumes are reported as percentage of total intracranial volume (% ICV). As a quick check of the validity of the automated volumetry, we compared volumes of the CA1, CA3, and CA4 subregions between patients with HS ILAE type 1 and ILAE type 2 (14), expecting more pronounced atrophy of the CA1 region in ILAE type 2. For further analysis, the ratio of the total hippocampal volume was calculated by dividing the total hippocampal volume of the surgical side by the non-surgical side. This ratio appeared larger than 1 for some people, indicating a larger hippocampus on the surgical side. To be able to perform correlation analysis, we also computed the normalized ratio of the total hippocampal volume, by dividing the smallest hippocampus by the largest hippocampus, irrespective of the surgical side.

### Intra-Operative ECoG Recordings

IoECoG was recorded using 2 × 4, 4 × 5, or 4 × 8 electrode-grids placed directly on the anterior laterotemporal cortex and one 1 × 8 electrode-strip placed subtemporally over the entorhinal cortex of the parahippocampal gyrus toward the hippocampus (Figure 1). The grids and strips (Ad-Tech, Racine, WI, USA) consist of platinum electrodes, embedded in silicone, with a contact surface of 4.2 mm^2^ and an inter-electrode distance of 1 cm. Recordings were made with a 64 channel EEG system (MicroMed, Veneto, Italy) at 2,048 Hz sampling rate with an anti-aliasing filter at 538 Hz. The signal was recorded referenced to an external electrode placed on the mastoid. Propofol was used as an anesthetic during surgery and was interrupted during recording until a continuous ioECoG background pattern was achieved. The ioECoG was repeated after the resection. Only the pre-resection ioECoG recording, sampling the anterior temporal pool plus hippocampus, was used for analysis.

### HFO Analysis

The last minute of ioECoG recording was selected for analysis to diminish propofol effect and artifacts. HFOs were visually marked by one reviewer (PA) and checked by a second reviewer (MZ). Marking was performed in Stellate Harmonie Reviewer in a bipolar montage. The display was split vertically with an 80 Hz high-pass filter and an amplitude of 5 μV/mm on the left side and a 250 Hz high-pass filter and 1 μV/mm on the right side. Ripples and fast ripples were marked if they clearly stood out from the baseline and contained at least four consecutive oscillations (15). An event was considered a prolonged ripple if there existed a clear oscillatory event lasting between 200 and 500 ms on the ripple screen (10). Rates of ripples, fast ripples and prolonged ripples were divided between subtemporal, if located on the first three channels of the strip, and neocortical, if located on other channels. Rates were calculated as the total number of events per channel divided by the total number of analyzed subtemporal, respectively, neocortical channels for each patient. The rates (events/minute) of HFOs were used for further analysis.

### Cognitive Assessments

Routine neuropsychological evaluation was performed in the year before surgery to assess the pre-surgical cognitive functioning of the patients. Standardized intelligence and cognitive tests, according to the age of the patient were applied. The Dutch versions of the Wechsler Intelligence Scale of Children III (WISC-III) (for children between 6 and 15 years) and the Wechsler Adult Intelligence Scale III (WAIS-III) (for patients age 16 or older) were administered by a clinical neuropsychologist to assess the total intellectual quotients (IQ), verbal IQ and performal IQ.

### Statistical Analyses

A non-parametric Wilcoxon signed rank test was used to compare the volume of each hippocampal subregion between surgical and non-surgical side and to compare HFO rates between subtemporal and neocortical channels. Mann-Whitney U tests were used to test for differences in hippocampal ratios and HFO rates between HS and non-HS patients. We compared localization of epilepsy in dominant or non-dominant hemispheres to IQ scores and HFO rates (Mann-Whitney U). We used a Spearman's Rho test for correlations between HFO rates and hippocampal volumes and HFO rates and IQ scores. Hippocampal volumes and IQ scores showed a normal distribution, so differences between ILAE type 1 and type 2 volumes were assessed with an independent sample *t*-test, and correlation between the hippocampal volumes and IQ was assessed with a Pearson correlation test. *P*-values <0.05 were considered to indicate statistical significance. We did not correct for multiple comparisons because this study is exploratory in nature and most comparisons are complementary, and sensibly planned based on hypotheses arising from existing evidence. Statistical analysis was performed in IBM SPSS Statistics 25 (IBM Corp., Armonk, NY).

## Results

### Population

Sixty-two patients diagnosed with MTLE had surgical resection of the hippocampus with ioECoG with grid and strip electrodes recorded at 2,048 Hz between 2008 and 2017. Thirteen patients had to be excluded from analyses because they presented dual pathology [FCD and HS (*n* = 5), cavernoma and HS (*n* = 3), glioma and HS (*n* = 3), Sturge-weber syndrome and HS (*n* = 1), glioneuronal tumor, ganglioglioma and FCD (*n* = 1)]. Two patients were excluded because of the presence of burst suppression in the epochs. Analyses were performed in 47 patients with an average age of 28 (range 2–62 years), of whom were 26 female. Nineteen patients underwent left temporal lobectomy (40%). Pathology results showed 10 patients with HS, 16 with a CNS tumor, four with a malformation of cortical development, 11 with other abnormalities and six without abnormalities (Table 1). *Forty-four* patients were right-handed, 28 had one or more language lateralization investigations (Wada *n* = 18, fTCD *n* = 14, fMRI *n* = 14), including all three left-handed patients. One left-handed patient had a right dominant hemisphere, one left-handed patient and two right-handed patients had bilateral language localization (based on fTCD + fMRI). The other 24 patients who underwent language lateralization were left dominant. Twenty patients had a dominant hemisphere surgery, assuming all right-handed patients without Wada, fTCD or fMRI were left dominant.

**Table 1:** Patient demographics.

	**HS**	**Non-HS**			
		**CNS tumor**	**MCD**	**Other**	**No abnormalities**
No.	10	16	4	11	6
Gender (No. female)	6	9	2	6	3
Age at surgery [Mn (range)]	26 (12–59)	15 (4–53)	49 (19–62)	40 (12–61)	32 (8–47)
Age at onset [Mn (range)]	6 (0–12)	9 (0–39)	20 (1–48)	20 (1–56)	18 (4–39)
Surgical side (L:R)	6:4	5:11	2:2	3:8	3:3

*HS, hippocampal sclerosis; CNS, central nervous system; MCD, malformation of cortical development; L, left; R, right; Mn, mean*.

### Hippocampal Volumetry

Six patients had a tumor located in or close to the hippocampus, which made reliable segmentation of hippocampal subregions impossible. These six patients were excluded in volumetry statistics. For the remaining 41 patients the total hippocampus on the surgical side was significantly smaller than the hippocampus on the nonsurgical side (median 0.22 vs. 0.23%, *Z* = −2.57, *p* = 0.01). When splitting into subregions, the CA1, CA3, CA4, hippocampal tail, subiculum, GC-ML-DG, fimbria, and HATA were smaller on the surgical side.

**Figure 2:**
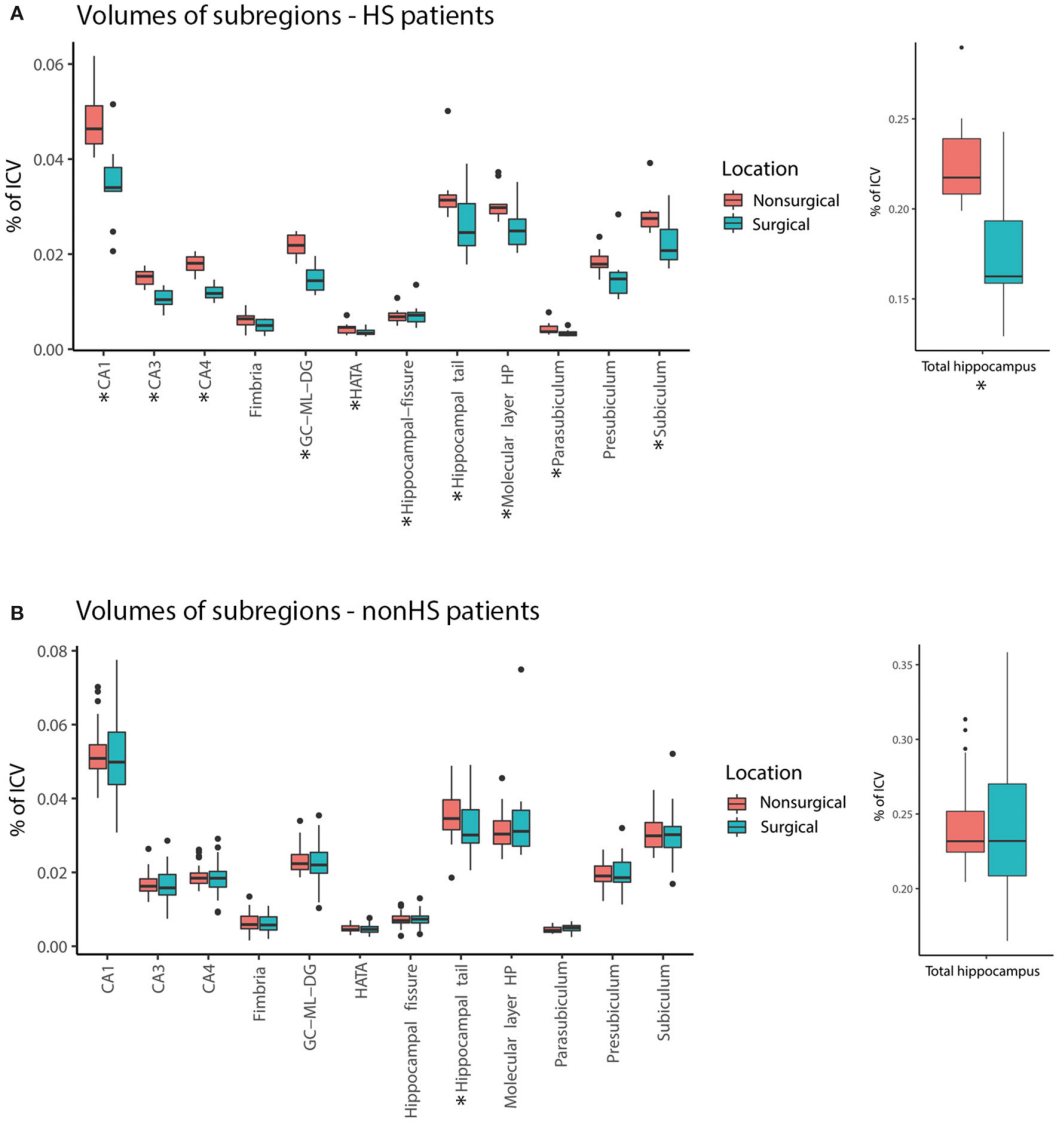
Comparison of volume [in % of total intracranial volume (ICA)] of hippocampal subregions surgical (blue) and nonsurgical (pink) hemispheres for hippocampal sclerosis patients (HS) **(A)** and non-hippocampal sclerosis (non-HS) patients **(B)**. The total hippocampus and many subregions were smaller in the surgical hemisphere compared to the nonsurgical hemisphere in the HS group, but not in patients without HS (* = statistically significant, *p* < 0.05).

In HS-patients, the total hippocampus on the surgical side was significantly smaller compared to the nonsurgical side. All subregions except for the presubiculum and fimbria were significantly smaller on the surgical side (Figure 2A). In patients without HS, there was no significant difference in total hippocampal volume between the surgical and nonsurgical side. Only the hippocampal tail was significantly smaller on the surgical side than on the nonsurgical side (median 0.03 vs. 0.04%, *Z* = −2.19, *p* = 0.028, Figure 2B).

Eight out of the 10 hippocampal sclerosis patients were ILAE type 1, the other two were ILAE type 2 (CA1 predominant). The mean volumes of the CA1, CA3 and CA4 areas were all non-significantly smaller in type 2 than in type 1 HS, but the difference was most prominent in CA1 [0.036% of ICV vs. 0.027% of ICV, t (8) = 1.50, *p* = 0.17] and CA3 [0.011% of ICV vs. 0.0086% of ICV, t (8) = 2.02, *p* = 0.08]. The difference in mean volume of the CA4 area was less pronounced (type 2 HS volume was 91% of type 1 HS volume).

**Figure 3:**
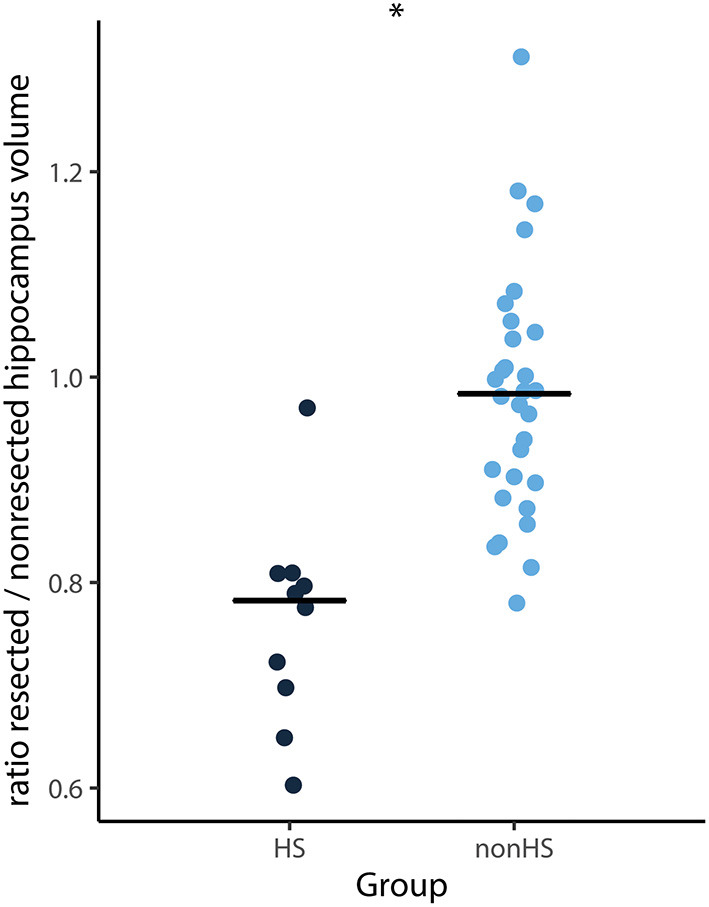
Ratio of total hippocampal volume (surgical side divided by non-surgical side) of each patient and median (horizontal lines) of HS (black) and non-HS (blue) patients. The ratio was significantly smaller for patients with HS compared to non-HS patients (*p < 0.001).

The ratio of the total hippocampus of the resected hemisphere divided by the non-resected hemisphere was on average 0.9. This ratio was lower for HS compared to non-HS patients (median 0.78 vs. 0.99, *Z* = −4.19, *p* < 0.001) (Figure 3). Twenty-one of 41 patients had a resected hippocampus that was more than 10% smaller than the non-resected hippocampus. Seven patients had a resected hippocampus that was more than 10% larger than the non-resected hippocampus. Three of them showed tumor mass, one had an MCD, two had other pathology and in one pathology showed no abnormalities.

### HFO Analysis

A total of 835 bipolar channels (657 grid and 178 strip) was analyzed (mean 18 (range: 10–35) per patient). A total of 1598 ripples (*n* = 37, mean 6.0 channels with events per patient), 259 fast ripples (*n* = 22, mean 3.2 channels with events per patient) and 285 prolonged ripples (*n* = 23, mean 2.8 channels with events per patient) were identified. Nine patients showed no HFOs at all, an additional 15 patients showed no fast ripples. One patient showed only prolonged ripples. Fast ripples were located only subtemporal, only lateral neocortical or both subtemporal and lateral neocortical in eight, eight and six patients respectively. Ripples were located only subtemporal in seven, only lateral neocortical in 10 and both subtemporal and lateral neocortical in 20 patients. Six of the 10 patients with HS showed subtemporal fast ripples and seven showed subtemporal ripples.

**Figure 4:**
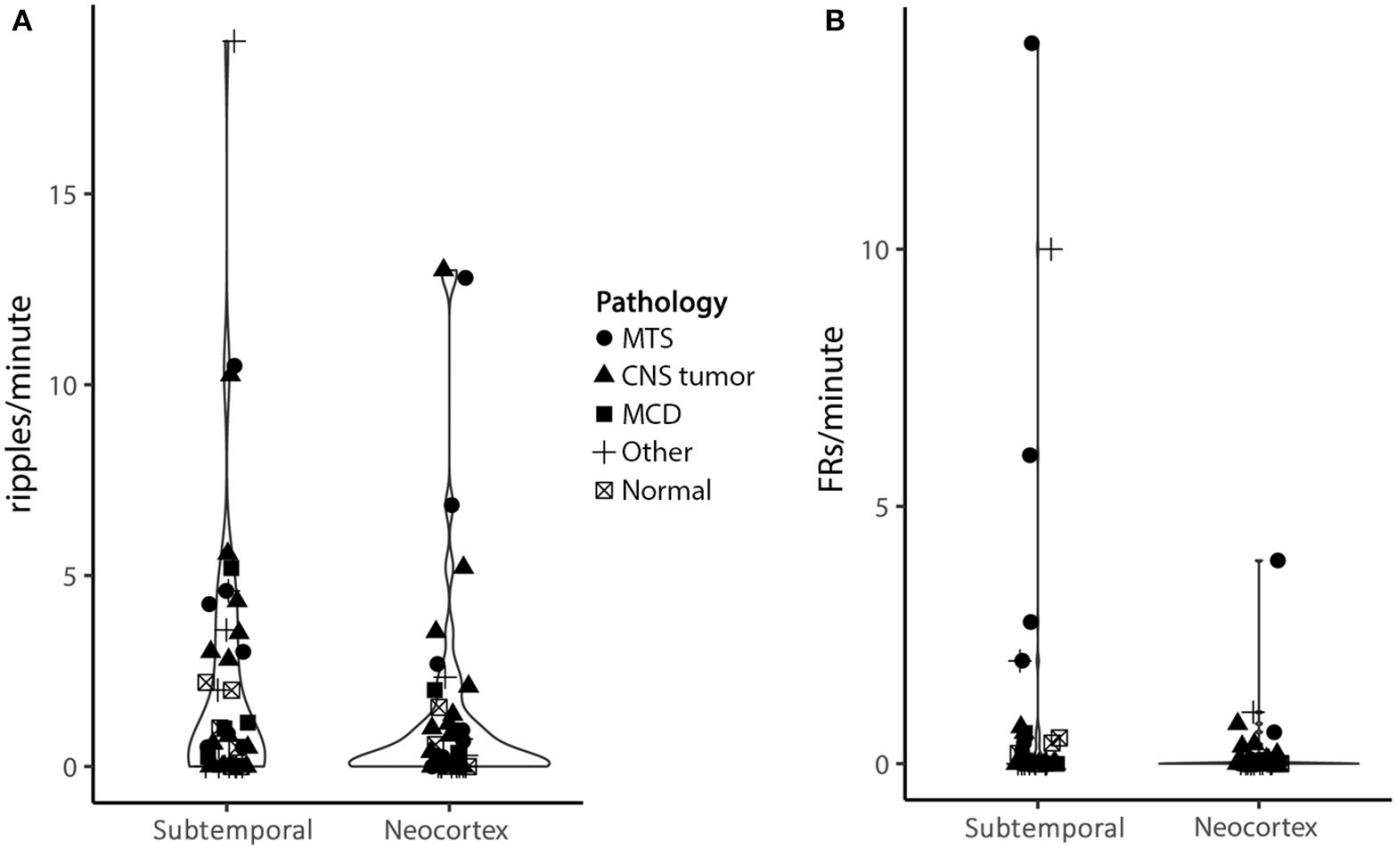
Violin plot of ripple **(A)** and fast ripple **(B)** rates in subtemporal and neocortical channels. Each character represents a patient, different characters represent different pathologies. The width of the violin represents the number of dots at a certain y value. Although not significant, there is a trend toward higher HFO rates in the subtemporal compared to the neocortex (ripples: 2.1/min vs. 1.3/min, *Z* = −1.28, *p* = 0.20; fast ripples: 0.9/min vs. 0.2/min, *Z* = −1.74, *p* = 0.08) (MTS, mesiotemporal sclerosis; CNS, central nervous system; MCD, malformation of cortical development).

There seemed to be a trend toward higher HFOs rates in the subtemporal compared to neocortical channels (ripples: 2.1 vs. 1.3/min, *Z* = −1.28, *p* = 0.20; fast ripples: 0.9 vs. 0.2/min, *Z* = −1.74, *p* = 0.08; Figure 4). Prolonged ripples were only found in the lateral neocortical channels.

HFOs and HS Both HS patients and non-HS patients showed fast ripples in the subtemporal channels, but the rate in non-HS patients was so low that the median rate was 0.0/min. The fast ripple rates in the subtemporal channels in HS patients were significantly higher than in non-HS patients (median 0.3 vs. 0.0/min, *Z* = −2.51, *p* = 0.012).

**Figure 5:**
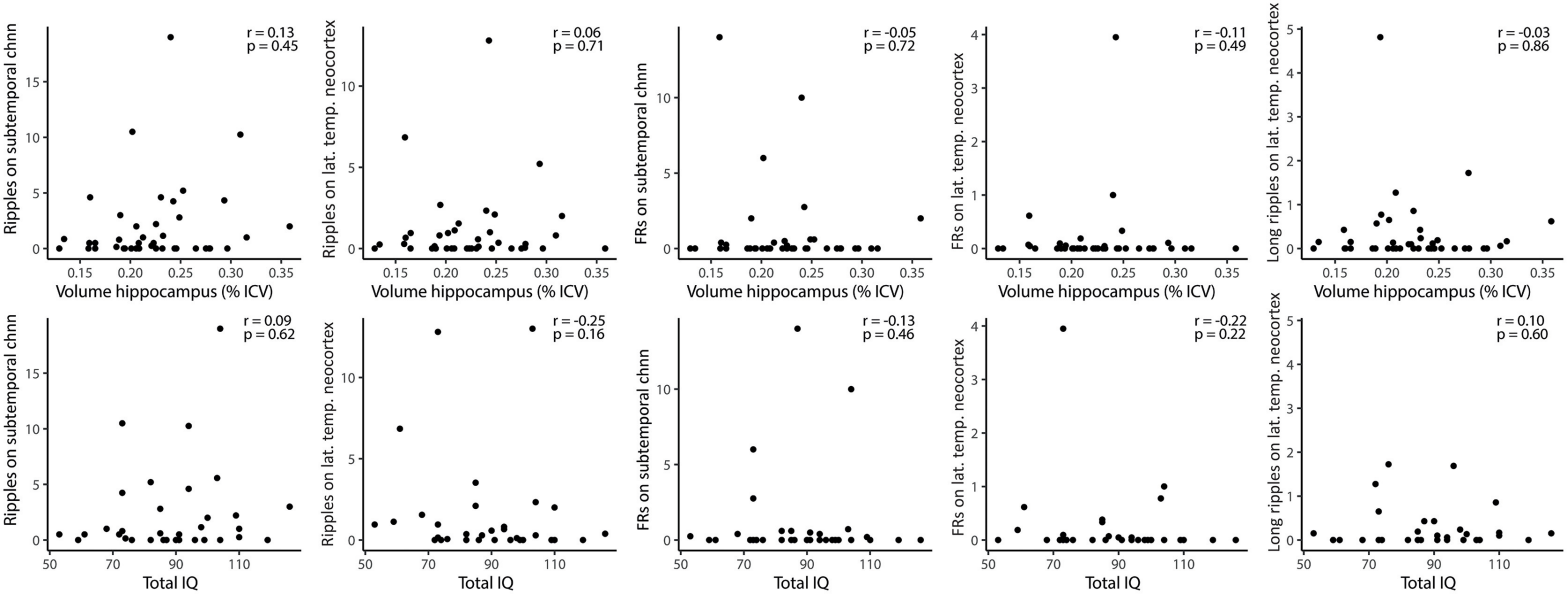
Correlations between HFO rates and volume of resected hippocampus (upper row), and HFO rates (subtemporal and lateral temporal neocortical channels) and total pre-resection IQ (lower row). None of the Spearman correlations was significant. FRs, fast ripples; Chnn, channel; lat., lateral; temp., temporal.

HFOs and hippocampal volume There was no significant correlation between lateral neocortical or subtemporal ripple, fast ripple, or prolonged ripple rates and the total volume or any of the subregions of the removed hippocampus or the (normalized) ratio of total hippocampal volumes (Figure 5). When specifically looking into patients with HS (*n* = 10), there was no significant correlation between total hippocampal volume and fast ripple rate in subtemporal channels (Spearman's *r* = 0.44, *p* = 0.21), nor between any of the subregions and subtemporal ripple or fast ripple rates. There was also no difference between the presence of lateral neocortical or subtemporal ripples or fast ripples (yes vs. no) and the total volume of the resected hippocampus or the (normalized) ratio of total hippocampal volumes.

### Intellectual Coefficient

Total IQ was available in 33 patients who underwent routine pre-surgical neuropsychological assessment. Twenty-two of them also had the verbal and performal IQ reported. Total, verbal and performal IQ did not differ between dominant hemisphere epilepsies and non-dominant hemisphere epilepsies (total IQ *p* = 0.16; verbal IQ *p* = 0.74 and performal *P* = 0.41). Hemispheric dominance did not yield different subtemporal or lateral temporal neocortical ripple, fast ripple or long ripple rates.

**Figure 6:**
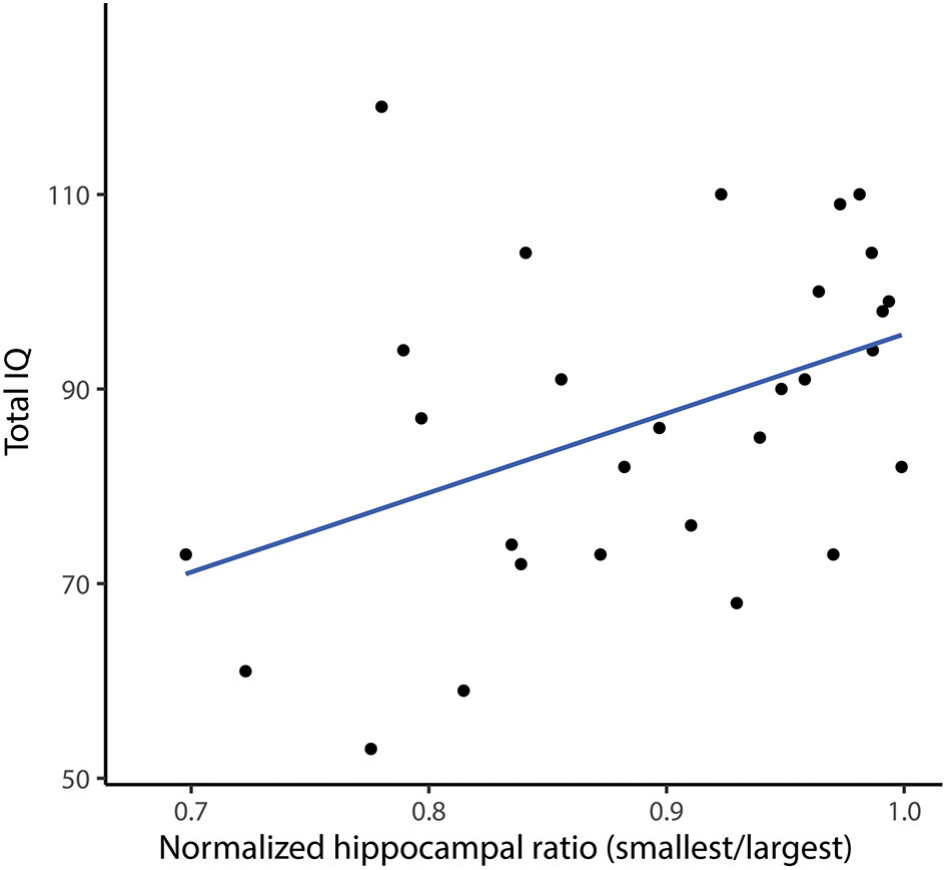
Correlation between pre-resection total IQ and normalized hippocampal ratio (smallest/largest). Each dot represents a patient, the blue line is the fitted linear regression line. There is a positive correlation between pre-resection total IQ and normalized hippocampal ratio, indicating a lower IQ when the left-right hippocampal volume difference is lager (Spearmans *r* = 0.45, *p* = 0.015).

The total IQ showed a significant correlation to the normalized ratio of total hippocampal volume (*r* = 0.45, *p* = 0.015, Figure 6), indicating a lower IQ in patients with a lower normalized ratio, and therefore a larger left-right difference of hippocampal volume. The pre-surgical verbal IQ showed the same significant correlation (*r* = 0.51, *p* = 0.024), while the pre-surgical performal IQ showed no significant correlation (*r* = 0.29, *p* = 0.23). Total, verbal and performal IQ did not differ between dominant hemisphere epilepsies and non-dominant hemisphere epilepsies (total IQ *p* = 0.16; verbal IQ *p* = 0.74 and performal IQ *p* = 0.41). There was also no difference in pre-surgical total IQ between patients with a right or a left sided temporal resection.

The total IQ showed a non-significant trend toward a positive correlation with the volume of the removed hippocampus (*r* = 0.32, *p* = 0.095). There was no significant correlation between neocortical or subtemporal ripple, fast ripple, or long ripple rates and total IQ (Figure 5). Hemispheric dominance did not yield different subtemporal or neocortical ripple, fast ripple or long ripple rates.

## Discussion

Patients with HS showed higher rates of subtemporal fast ripples than other patients. We found no relation between HFO rates and hippocampal volumes or IQ. We found a trend toward higher rates of HFOs in the subtemporal channels compared to the neocortex, and significantly higher fast ripple rates in the subtemporal channels in patients with HS. Prolonged ripples were only found in the neocortex. Patients with a large left-right difference in hippocampal volumes had a lower pre-surgical IQ.

As expected, our data showed volume reduction of the ipsilateral total hippocampus in HS patients, supporting the results from other studies (12, 16–18). This volume reduction was present in almost all subregions (CA1, CA3, CA4, hippocampal tail, subiculum, GC-ML-DG, fimbria and HATA), in agreement with previous studies (12, 18, 19). The distribution of atrophy is in line with the typical volume loss pattern described by histopathological studies (1, 18). We found only atrophy of the hippocampal tail in a subselection of non-HS patients. Interestingly, in 17% of the patients (all non-HS; three with tumors, one MCD, two other and one without pathological abnormalities) the resected hippocampus was >10% bigger than the non-resected hippocampus. An explanation for a bigger hippocampus on surgical side could be ipsilateral swelling, for example due an subtle underlying pathology (e.g., FCD or tumor), or contra-lateral atrophy, as has been described for the amygdala in MTLE without HS (20, 21). We did not analyze amygdala volumes in this study. We found a lower pre-resection IQ was associated with a lower total volume of the resected hippocampus, which was expected as both worsen with longer duration of epilepsy (22).

Fast ripples arose at a higher rate in the HS- vs. non-HS patients. This is in line with previous studies that found higher rates of fast ripples in patients with hippocampal sclerosis (15, 23, 24). Even though the mechanisms underlying the generation of HFOs are still unclear (6), it is suggested that this is due to excitotoxicity occurring in HS (25). *Ex vivo* studies have found high levels of extracellular potassium (K^+^) in the sclerotic hippocampal tissue generating fast ripples, but no fast ripples were found in a non-HS group with the same levels of K^+^ (26). Neuronal loss would interrupt the recapture pathway of K^+^, leading to an accumulation of K^+^ in extracellular spaces that influence neuronal excitability and high frequency neuronal activity in the sclerotic hippocampus (26).

HFO rates did not correlate with hippocampal volumes. This is in contrast with other studies that found an association between fast ripples rate and atrophy (25) or fast ripples to ripples rate ratio and atrophy (27) with the degree of hippocampal atrophy. Our data shows that the fast ripple rate in the subtemporal channels is higher in case of hippocampal sclerosis, but the rate was not linearly related to the amount of atrophy. We know that HFOs are related to the seizure frequency at that time point (28). Seizure frequency is not necessarily related to the amount of atrophy at that time point, but mainly to the duration of epilepsy (22). We do not have information about current seizure frequency of this cohort but this could explain our findings. The reason for the discrepancy with previous literature might also be the difference in the recording methods. We used subtemporal strip macro electrodes to sample the entorhinal cortex which covers the hippocampus, while both studies that found a correlation used micro-electrodes stereotactically inserted in the hippocampus. Worrell et al. (23) compared HFO rates recorded with micro- and macroelectrodes and hypothesized that these differences in ripples and fast ripples rate are due to the spatial undersampling of focal HFO activity with macro-electrodes. Our study included only 10 patients with hippocampal sclerosis, which might be too small to show a relation between hippocampal volume and HFO rates.

Recent research performed on sEEG recordings, has suggested that continuous rippling (with a longer duration > 200 ms) found in mesiotemporal and occipital areas is independent of epileptogenicity as they do not correlate with the seizure onset zone, lesions or surgical resection area. Thus continuous rippling might reflect a particular type of physiological discharge (7–10). The hippocampus above all other structures typically generates physiological ripples, which are involved in memory consolidation, and their occurrence is strongly linked to neocortical slow waves during natural sleep (29, 30). Although propofol anesthesia is a sleep-like state that also shows slow waves, these waves are, in comparison to natural sleep, more spatially blurred and without spindling in comparison to natural sleep (31).

We marked prolonged ripples in an attempt to differentiate between physiological and pathological ripples. We found prolonged ripples only in lateral temporal, neocortical, channels. This is in contrast with other sEEG studies, that found physiological ripples in presumed normal hippocampi (29). Earlier studies have shown that differentiation between individual physiological and pathological ripples based on duration alone is not adequate (30, 32, 33), but our hypothesis was that the majority of the prolonged ripples would be physiological. The fact that we did not record prolonged ripples from the hippocampus means either that physiological ripples were not prolonged, or the hippocampus did not produce physiological ripples due to the surgical circumstances including administration of propofol before the recording. It is remarkable in this context that we do not remember seeing the typical pattern of continuous ripples in the hippocampal areas that can be seen in sEEG recordings (7). We did not see this in our intra-operative data, neither in this study, neither in previous studies nor in the onsite intra-operative review of HFOs for the HFO trial (34). We are used to seeing prolonged ripples in neocortical grid electrodes covering Broca's area, the central area and occipital area. This difference from sEEG recordings may result from the surgical conditions and would be interesting to study in more detail in the future.

We chose IQ as measure for cognitive function, even though hippocampal pathology affects memory most specifically. We did this because of the wide age range and diversity in testing, which always included an IQ score but not always a numerical memory score. IQ gives the measurement of the patient's general cognitive functioning and can be corrected for age. It has been demonstrated that patients with MTLE not only encounter memory deficits, but also impairment in all their cognitive functions (11). We recently showed that children in whom the area showing fast ripples on ioECoG was removed, had better chance at IQ improvement after surgery, irrespective of seizure recurrence (35). To date studies have only found the relationship between high HFO rates with memory impairment in MTLE (36, 37) while the role of HFOs in overall cognitive functioning have not been documented yet. In this study we could not confirm the relation between HFO rates and cognitive functioning. Since we do not have a cohort with MRIs of control subjects without epilepsy, we could not quantify the amount of atrophy compared to a normal hippocampus. When patients have bilateral atrophy, this will also affect the ratio of the hippocampal volume, which will be closer to one the more equally both hippocampi are affected. We tried to minimize this effect by focusing most on between-subject analyses on the volume of the resected hippocampus, corrected by total intracranial volume.

The use of intra-operative ECoG recordings has several limitations for data analysis and interpretation. First, in contrast to extra-operative recordings, intra-operative recordings are usually 3–4 min, of which the first minutes are often contaminated by burst suppression (38–40). Availability of epochs longer than 1 min might have resulted in different HFO rates with especially more chance to capture fast ripples. Second, intra-operative ECoG recordings are limited to recording the presumed affected hippocampus, making it impossible to compare HFO rates between hippocampi. Third, we used the HFO rates on the first three channels of the subtemporal strip recording the entorhinal cortex of the parahippocampal gyrus as a proxy for the hippocampus. We considered the hippocampus to be the source of events observed on the first three channels of the subtemporal strip, because on these channels typical hippocampal spikes, similar to those in sEEG, can be seen. At least part of the signal however arises from the overlapping entorhinal cortex, which can also show HFOs and atrophy, but is often secondarily to hippocampal atrophy (41). This could explain why we did not find a correlation between hippocampal volume and subtemporal strip HFOs. We did not analyze the volume of the entorhinal cortex as it was often affected by the epileptogenic lesion. SEEG records directly from within hippocampi and the electrode positions are verified by MRI. It would be of interest to investigate how these intra-operative HFO rates relate to extra-operative HFO rates in the same patient. To conclude, we found increased fast ripple rates on the subtemporal channels in ioECOG in patients with HS, but ripple, fast ripple or prolonged ripple rates did not correlate with the hippocampal volume nor with IQ. We found prolonged ripples only in neocortical but not in subtemporal channels, and they were not related to IQ or volume reduction. Further research is needed to understand prolonged ripples and their role played in epilepsy and cognition.

## Data Availability Statement

The raw data supporting the conclusions of this article will be made available by the authors, without undue reservation.

## Ethics Statement

The studies involving human participants were reviewed and approved by METC Utrecht. Written informed consent from the participants' legal guardian/next of kin was not required to participate in this study in accordance with the national legislation and the institutional requirements.

## Author Contributions

PA, NK, MK, BS, WZ, TG, MZa, PE and MZi in collaboration with the RESPect database study group were involved in data collection. PA, NK, WZ, and MZi were responsible for the design of the study, and the HFO analysis. PA and NK performed the statistical analysis. PA, NK, MK, and MZi drafted the manuscript. MK, PA, and NK prepared the figures. All authors were involved in review, editing, and approval of the final version of the manuscript.

## RESPect Database Study Group

Dorien van Blooijs, Kees Braun, Matteo Demuru, Cyrille Ferrier, Peter Gosselaar, Geertjan Huiskamp, Frans Leijten, Janine Ophorst, Peter van Rijen, Sandra van der Salm, Anouk Velders.

## Conflict of Interest

The authors declare that the research was conducted in the absence of any commercial or financial relationships that could be construed as a potential conflict of interest.
